# Still in search of a herbal medicine…

**DOI:** 10.4103/0253-7613.48876

**Published:** 2009-02

**Authors:** Urmila Thatte

**Affiliations:** Dept. of Clinical Pharmacology, Seth GS Medical College and KEM Hospital, Mumbai 400 012, India. E-mail: urmilathatte@kem.edu

As an undergraduate medical student, the first aspect of pharmacology that fascinated me was how plants had been exploited for their medicinal potential. From aspirin to digitalis and morphine to reserpine, the stories of their discovery to development captured my imagination. This fascination extended into my postgraduate days and, in retrospect, was also perhaps responsible for goading me into working on medicinal plants for my doctoral research. The development of *Tinospora cordifiolia* under the leadership of Dr. Sharadini Dahanukar from “stem to pill” has been a topic of much discussion, but underpinning that success, were many challenges and obstacles, some of which we were able to address while others still continue to trouble.

Almost two decades after our first publication on *Tinospora cordifolia*, herbal research still remains an area of interest for many a pharmacology department as is evident from my small study of articles published in the Indian Journal of Pharmacology over the last two years. Out of a total of 67 articles (excluding editorials, correspondence and review articles) published in this period, 44 were on plants. Although primarily coming from pharmacy and medical schools, some were also from Biological and Animal Science departments. These papers evaluated almost as many plants as there were papers with only two plants being studied more than once - *Morus alba* thrice and *Embelia ribes* twice. Most researchers used aqueous extracts (13 papers), while ethanolic (11 papers) and methanolic (8 papers) extracts followed in popularity. Other test materials ranged from hydroalcoholic to petroleum ether extracts to “actives” and flavonoids. Most researchers have studied the effects of leaves (17 of 44), while 4 used the whole plant, 5 described fruits and only 3 described effects of roots. From the point of view of drug development, both these aspects are important. If we were to consider these reports as leads and would want to take off from this point, working on extracts other than aqueous or hydroalcoholic would involve the complete and often tortuous and expensive path of drug development that a new chemical entity (NCE) would follow (see below). Similarly, if source of drug is the whole plant or root, the continued availability of raw material of standardised and acceptable quality becomes a genuine challenge from the point of view of sustainability and environmental conservation which has to be kept in mind while choosing the part of plant to study.

The relevance of research to our healthcare problems is another important aspect. Although 44 papers published in the IJP is not a large sample size (and importantly does not include the work done toward papers that were rejected), it still represents the top layer of work done across the country. A quick look at the activities tested reveals a very wide spectrum. While anti-diabetic activity was studied in 6 articles and anti-oxidant in 4, all other articles have reported completely disparate actions ranging from anti-anxiety and anti-asthma to anti-malarial. When I looked at the 6 papers describing anti-diabetic effects of various plants, I found that they primarily concentrated on hypoglycaemic effects. Experimental models included glucose overloaded hyperglycemic rats,[1] alloxan induced diabetes,[2,3] streptozotocin + nicotinamide induced diabetes[4], and streptozotocin (alone) induced diabetes models.[5] All plants under investigation produced significant hypoglycaemia – however, the question is do we need a hypoglycaemic agent or should we be looking at other issues (such as complications) in diabetes? Should insulin sensitisation or beta cell conservation be studied, for example?

This very brief analysis highlights the major problems that beset herbal drug development, perhaps hinting at the reason that in spite of increasing research in herbal medicines, (almost by 58.9% from 1995 to 2003 – as revealed in a letter to Editor published in the IJP in 2004)[6] there is still no product, no drug, that has made a difference to therapeutics.

Many reasons can be found for this and the challenges of research on herbal medicines have been discussed in the past.[7] The plant itself is the source of the biggest challenge and the correct choice underpins the beginning of success. From identification, to method and site of harvesting, the part being used, the processing, standardisation, purity and the final formulation, all have to be taken into consideration.[8] Special attention must also be paid to standardisation of the compound, using markers (if possible, bio-active markers). Although most publications mention that “the extract was authenticated and standardised”, the information is inadequate from a drug development point of view. Far more details are required to be furnished[9] and generation of this information is crucial to further studies. Naturally this work has to precede the experimental or clinical evaluation to allow the use of a really standardised product (against the predefined specifications for the raw material, extract and also the final formulation for clinical use). Much work does happen in various pharmacognosy departments, but the compartmentalisation of expertise has prevented the development of fruitful collaborative programs leading to successful drug development.

In conventional drug development, the safety (and toxicity, or lack thereof) of the chemical is what influences whether the chemical becomes a medicine. In the case of plants (especially those used in traditional medicine) this topic has been much debated. How much toxicity testing is required? The ICMR Guidelines[10] have classified the plants into three categories: Category I includes those plants or extracts about which a lot is known in ancient literature or the plant may actually be in use by physicians of the traditional system of medicine. In such cases, the extent of toxicity testing required is less. When an extract of a plant or a compound isolated from the plant has to be clinically evaluated for a therapeutic effect not originally described in the texts of traditional systems, or, the method of preparation is different from tradition, it is described as belonging to Category II. It is recommended that this type of extract has to be treated as a new substance or a new chemical entity (NCE) and the same type of acute, subacute and chronic toxicity data has to be generated as required by the regulatory authority for synthetic products before it is cleared for clinical evaluation (although here it is important to note that at least at present there is much ambiguity about who is the Licensing Authority for permitting clinical trials on herbal products). Category III are new extracts and they are also treated as NCEs. Surprisingly a large number of papers published in IJP over the past two years (on herbal medicine) reported acute toxicity data raising the question whether utilizing scarce resources on LD50 studies on plants extracts was appropriate.

Moving from the experimental scene to the clinic is important in drug development. Despite the existence of the concept of reverse pharmacology,[11] it was discouraging that in the last two years, only 3 of the 44 papers on plants published in the IJP were clinical studies.This is probably because the issue of clinical testing opens a Pandora's box, as designing a clinical study with herbal drugs is an exercise in itself. Several issues have to be addressed, including the sample size, the study design, controls, the inclusion and exclusion criteria and the choice of efficacy and safety variables and end points. The gold standard of clinical research is the randomised, controlled clinical trial design. Such studies are often difficult with herbal medicines. What comparator to use? Do we really expect herbals (which are at best a mixture of multiple chemicals in concentrations that are not really known when the study is initiated) to work as well as or better than the pure chemicals (like metformin in diabetes or anti-cancer drugs in malignancies)? The choice of placebo has also to be made carefully– to match the colour, flavour and consistency of the herbal material is challenging. While determining the inclusion criteria, it appears necessary to consider such factors as the *prakriti* (or constitution of the participant),[12] as this can influence the drug response. Sometimes because of scepticism or overenthusiasm, patients who are not responding to “conventional medicine” are included in the study. These studies are unfair to the herbal medicine, as with this eligibility criterion, the chances of success of any (not just the herbal) therapy become low. Selecting “hard” end-points and variables for clinical evaluation of herbal medicines is often disappointing, especially with the medicines showing up to be no more “effective” than placebo. However, when compared using “symptom complexes” as described in traditional medicine there is often a response seen. Apart from these considerations regarding study design, the plant material with which to do the clinical trial does also need special attention. This is particularly relevant when long term studies are planned where shelf life and stability of the formulation need to be ensured as also the packaging and transport of this otherwise perishable material. While choosing the correct dose for clinical studies, extrapolation of a dose recommended in the traditional medicine proves a useful approach, as with most of the herbs pre-clinical data (in terms of both safety and efficacy) is not available and more importantly, pharmacokinetic data is missing completely. The formulation, another tricky issue, should be selected carefully. Ethical issues also raise dilemmas. One has to be vigilant while documenting informed consent in studies with herbal drugs. It must be assured that patients are not opting for these remedies because of deep-rooted beliefs in their safety! Further it should be kept in mind that patients have easy access to marketed or home remedies that are usually not considered as medication by them and if consumed may confound results!

Perhaps because of these challenges we have not seen too many publications on clinical trials with herbal medicines globally. Quality of the published trials has also been questioned.[13] Evidence to justify the use of herbal medicines in mainstream medicine is therefore missing.[14] A search of Cochrane Reviews for herbal medicines reveals more than 200 reviews on herbal therapy for diseases ranging from AIDS, dysmenorrhea and even sore throat to angina pectoris and diabetes. A common refrain at the end of each review is “Although there appears to be some potential benefit, further studies are required, and the evidence remains weak due to poor methodological quality and small sample size”. What is remarkable is that almost all are reviews on Chinese medicines, none are on Indian herbs. This would probably be due to inadequate material for review – further substantiating the fact that there is very little clinical research published.

What needs to be done? A focused “drug development” approach to research is essential, particularly in academia. It is exciting to note that the Government of India, through its various programs has been encouraging focussed drug development. For example, the Department of Biotechnology has supported a multi-institutional programme to develop a standardized and safe herbal product from *Terminalia arjuna* for left ventricular dysfunction. Other areas where work is in progress include diabetes, amoebiosis and atherosclerosis. (http://dbtindia.nic.in/uniquepage.asp?id_pk=340; accessed 8th Feb. 2009). Similarly, under the CSIR's NMITLI programme (http://www.csir.res.in/csir/external/heads/collaborations/sa.pdf; accessed 8th Feb. 2009), anti-arthritic and anti-diabetic compounds have been developed while the Golden Triangle Partnership (http://www.ccras.nic.in/Golden_Tiangle/20081011_Golden_Tiangle1.htm; accessed 9th Feb 2009) promises exciting results to look forward to because of its “out of the box” approach to herbal research.

The final point worth pondering is why the pharmaceutical industry in India, barring a few isolated examples, is not considering herbal research seriously. Is it because of a perceived lack of IPR protection? I did a simple patent search on *Tinospora cordifolia* and found that 43 international patents have been granted for actions ranging from diabetes to “liver protective” and yet these have not been commercially exploited. In a conventional setting a hand shake between industry and academia forms the basis of new drug development. This is still not very evident in India in spite of a number of Government schemes to promote industry-academia interaction.

To summarise therefore, a national level focussed drug development program is required, led by competent and dynamic individuals, in areas prioritised to suit the country's health care needs (diabetes comes to mind as a good starting point). The key here has to be a strong inter-disciplinary team with a sense of partnership amongst all stakeholders (botanists, phytochemists, pharmaceutic experts, molecular biologists, pharmacologists, clinical pharmacologists and clinicians) and a strong commitment from the industry.
